# The rate of exacerbations in patients with asthma in Qatar: A retrospective study during 2019-2021

**DOI:** 10.5339/qmj.2023.sqac.22

**Published:** 2023-11-26

**Authors:** Dina Isaifan, Maryam Al-Nesf, Lama Soubra, Hassan Mobayed, M. Rami Alfarra, Sergio Crovella

**Affiliations:** ^1^Biological and Environmental Sciences Department, Qatar University, P.O. Box 2713, Doha, Qatar Email: di1510538@student.qu.edu.qa; ^2^Allergy and Immunology Division, Hamad Medical Corporation, P.O. Box 3050, Doha, Qatar; ^3^Qatar Environment and Energy Research Institute, Hamad Bin Khalifa University, P.O. Box: 34110, Doha, Qatar

## Abstract

**Background:** The prevalence of asthma is 9% among adults in Qatar, and its severity can be attributed to intrinsic and extrinsic factors such as environmental changes. As part of the project to investigate the association between air pollution and asthma severity, the rate of exacerbations in adult patients with asthma has been studied in Qatar.

**Methods:** Retrospective data of patients with asthma (16-70 years) from January 2019 to December 2021 was retrieved from Cerner medical records. Frequencies of exacerbations in inpatient and outpatient departments were analyzed using means ± SD and median (IQR) for descriptive data and frequency and percentage for categorical data. Exacerbations were divided into single, double, and more than double for each quarter of the year (January 2019-December 2021) using SPSS and Minitab statistical packages.

**Results:** A total of 6977 exacerbations visits (representing 6558 patients) were identified during the study period. The mean ± SD age was 41±14.3 years, with a female: male ratio of almost 1:1. The patients from the MENA region, including Qataris, presented 67% compared to 33% from the Indian subcontinent and other countries. The number of patient visits for hospitalization due to exacerbations showed a distinctive pattern during the three years. The highest record of asthmatics with exacerbations was observed in 2019 (42.7%) compared to half the rate in 2020 and 2021 (28.5%, 28.8%), respectively. The single exacerbation group was almost five times higher than 2 or >2 exacerbation groups in all years (2019-2021).

**Conclusion:** This preliminary overview provides the rate of exacerbation episodes in patients with asthma in Qatar. One cause of these exacerbations can be attributed to air quality changes. The drop in the exacerbation rate observed in 2020-2021 could be explained by COVID-19 lockdown regulations or patients’ adherence to prescribed meds. We aim to propose preventive and therapeutic strategies to alleviate asthmatics’ symptoms and improve their quality of life.
